# Calcium Carbonate (Tums)-Associated Hypercalcemic Crisis

**DOI:** 10.7759/cureus.32913

**Published:** 2022-12-25

**Authors:** Hanish Jain, Sindhubarathi Murali, Garima Singh, Ioana Amzuta

**Affiliations:** 1 Internal Medicine, Upstate University Hospital, Syracuse, USA; 2 Medicine, State University of New York Upstate Medical University Hospital, Syracuse, USA; 3 Internal Medicine, Saint Vincent Hospital, Worcester, USA; 4 Pulmonary and Critical Care/Sleep Medicine, State University of New York Upstate Medical University Hospital, Syracuse, USA

**Keywords:** acute hypercalcemia, milk alkali syndrome, gastroesophageal reflux disorder (gerd), over-the-counter, calcium alkali syndrome, calcium carbonate, hypercalcemic crisis

## Abstract

Hypercalcemic crisis due to severe decompensated hypercalcemia represents a life-threatening medical emergency leading to renal failure and altered mental status. Hypercalcemic crisis most commonly results from hypercalcemia of malignancy, undiagnosed primary hyperparathyroidism, granulomatous diseases, and medication-induced hypercalcemia. Commonly prescribed medications like thiazide diuretics, lithium therapy, and teriparatide are among the medications known to cause hypercalcemia. Here, we describe a case of the hypercalcemic crisis caused by excessive calcium carbonate ingestion emphasizing physicians to be aware of this potentially life-threatening adverse effect of a widely available over-the-counter acid reflux medication and educate patients regarding the same.

## Introduction

This article was previously presented as a meeting abstract at the 2022 CHEST conference on October 18, 2022.

In the early twentieth century, milk-alkali syndrome was commonly reported in men as a toxic adverse effect due to intensive treatment of peptic ulcer disease with calcium-containing antacids and milk [1]. Calcium carbonate is alkaline in nature and a major source of calcium and alkali [2]. Milk-alkali syndrome has been renamed ‘calcium-alkali syndrome’ as the previous name no longer indicates the etiology of the disorder. The disease is characterized predominantly by hypercalcemia, metabolic alkalosis, and renal failure [3].

## Case presentation

A 76-year-old Caucasian male with a history of diabetes mellitus on glipizide, hypertension on amlodipine and olmesartan, myelodysplastic syndrome on azacitidine, and gastroesophageal reflux disease (GERD) not on prescription medication was found lethargic and somnolent on the floor of his house. He was admitted to the Medical Intensive Care Unit with altered mentation. Vital signs showed pulse 104/ min, blood pressure 110/70 mm hg, RR 22/min, and saturating 96%. Physical examination was unremarkable except for dry oral mucosa. Given his encephalopathic state and risk of impaired secretions leading to aspiration, the patient required intubation to protect his airway. His routine lab work was normal except for an elevated calcium level of 17.4 mg/dl and creatinine of 4.27 mg/dl with a normal baseline of 0.6 mg/dl (Table 1). The patient received aggressive fluid resuscitation 2L NS bolus followed by maintenance fluid adjusted to maintain the urine output at 100 to 150 mL/hour, four doses of calcitonin four units/kg every 12 hours, and one dose of 60 mg pamidronate. He required vasopressor support secondary to the sedatives he received. The diagnostic workup for the patient's hypercalcemia showed an appropriately suppressed parathyroid hormone (PTH) response (decreased at 9 pg/ml) and a normal parathyroid hormone-related peptide (PTHrp) <2 pmol/L. ECG revealed sinus tachycardia and a corrected QT interval within the normal range. Imaging studies in the form of contrast computerized axial tomography scans of the brain, thorax, abdomen, and pelvis and brain magnetic resonance imaging ruled out a stroke or occult malignancy. Multiple myeloma was excluded based on normal serum and urine immunofixation studies. Prostate-specific antigen was normal. Urine analysis was unremarkable except random urine calcium was 18.2 mg/dL. There was a gradual decline in the patient’s serum calcium level over the next 48 hours while continuing to require continuous intravenous hydration with isotonic fluid. The calcium levels normalized after seven days. After getting extubated, the patient reported that he took 8-9 tablets of 1000 mg tums daily for a few days for his GERD before his hospital admission. After a thorough negative workup, the etiology of his hypercalcemia was deemed most likely secondary to the ingestion of calcium carbonate (tums). On post-hospital discharge follow-up, calcium levels remained normal, and the primary care provider started the patient on histamine blockers for his GERD.

**Table 1:** Laboratory characteristics during admission, hospital course, and clinic follow-up BUN, blood urea nitrogen; PTH, parathyroid hormone ^a^PTH: normal reference interval 10–65 pg/mL. ^b^25(OH)D: normal reference interval 30–100 ng/mL.

Lab values	Day 1	Day 2	Day 7	Day 7
Serum calcium (mg/dL)	17.4	12.2	9.4	8.6
Serum phosphorus (mg/dL)	3.8	2.8	2.4	2.0
BUN (mg/dL)	56	42	34	22
Serum creatinine (mg/dL)	4.27	1.4	1.26	0.74
Serum bicarbonate (mEq/L)	32	28	26	22
Intact PTH (pg/mL)^a^	9			
25(OH)D (ng/mL)^b^	38			

## Discussion

The classic triad of calcium-alkali syndrome consists of hypercalcemia, renal failure, and metabolic alkalosis due to the ingestion of large amounts of calcium and absorbable alkali [4]. The clinical presentation may vary depending on the severity of the hypercalcemia. The patient may be asymptomatic, dehydrated, or present with symptoms of intractable nausea, vomiting, constipation, and altered mental status. The severity of acute renal insufficiency is dependent on the level of serum calcium. Higher elevations in the serum calcium concentration (serum calcium values of 12 to 15 mg/dL) can lead to a reversible fall in the glomerular filtration rate that is mediated by direct renal vasoconstriction and natriuresis-induced volume contraction [5]. Most patients presenting with severe hypercalcemia have marked intravascular volume depletion. Hypovolemia exacerbates hypercalcemia by impairing the renal clearance of calcium. Isotonic saline for 24 to 48 hours corrects possible volume depletion due to hypercalcemia-induced urinary salt wasting [6]. The striking feature in our case was the severity of hypercalcemia in an elderly patient secondary to an excessive intake of calcium supplements which supports the diagnosis of calcium-alkali syndrome. Many cases of calcium-alkali syndrome resulted primarily from the consumption of calcium carbonate-containing antacids [3]. Usually, these were patients who had an underlying history of reflux esophagitis and dyspepsia. Calcium-alkali syndrome is also seen in individuals with hypertension exposed to diuretics, angiotensin-converting enzyme inhibitors, and angiotensin receptor blockers resulting from hypovolemia and calcium retention. Regarding the treatment of GERD, many providers have stepped away from prescribing proton pump inhibitors given their side effects such as acute interstitial nephritis, fractures, and Clostridium difficile-associated diarrhea. This has resulted in increased usage of calcium carbonate as an antacid and calcium supplement to help treat or prevent osteoporosis [7]. Over-the-counter medications are believed to be safe since hypercalcemia typically results after ingestion of more than four grams of calcium per day [8]. Previous studies have argued that certain patients are more susceptible to hypercalcemia, even when ingesting dosages below the daily limit recommended by the manufacturer [9]. 

## Conclusions

In conclusion, a life-threatening hypercalcemic crisis can be caused by over-the-counter calcium supplements. With expensive investigations, knowledge of such an adverse effect of calcium alkali syndrome is necessary to prevent further occurrences and unnecessary workup of malignancy and hyperparathyroidism. Physicians and surgeons should be mindful of such adverse effects, especially in a more vulnerable patient population suffering from gastroesophageal reflux disease. Clinicians should perform thorough medication reconciliation, including over-the-counter medications, which can help them educate patients on the risks associated with antacid ingestion.
